# Prevalence of Traumatic Dental Injuries to Anterior Teeth in 8–12-year-old Schoolchildren of Patiala City, Punjab, India: An Epidemiological Study

**DOI:** 10.5005/jp-journals-10005-1583

**Published:** 2019

**Authors:** Charan KK Dharmani, Anuradha Pathak, Haridarshan S Sidhu

**Affiliations:** 1 Dental Department, Government Multi Specialty Hospital, Sector-16, Chandigarh, India; 2,3 Department of Pedodontics and Preventive Dentistry, Government Dental College, Patiala, Punjab, India

**Keywords:** Body mass index, Cross-sectional study, Obesity, Overjet, Prevalence, Trauma

## Abstract

**Aims:**

The aim of this study is to determine the prevalence of traumatic dental injuries (TDIs) to anterior teeth in 8–12-year-old schoolchildren of Patiala city in North India and to study the risk factors related to dental trauma.

**Materials and methods:**

An epidemiological cross-sectional survey was conducted. The study consisted of 3,000 schoolchildren from various schools of Patiala city in North India. The body mass index was calculated. Lip competency, anterioposterior molar relationship, overjet, and overbite were examined. Dental injuries to anterior teeth were recorded according to the Ellis classification. Data were collected on a proforma.

**Results:**

The prevalence was found to be 11.4%. Maximum injuries occurred in the 11-year age group and the boys:girls ratio was 1.7:1. The home was the most common place with fall being the most common reason. The relationship between obesity and dental trauma was found to be statistically insignificant, whereas a statistically significant relationship was found between TDIs and incompetent lips, incisal overjet ≥5 mm, and Angle's class II div I molar relation. The Ellis class I fracture was the most predominant type.

**Conclusion:**

The high prevalence of dental trauma stresses the need for the development of preventive strategies. There is a need for increased awareness among parents and children regarding dental trauma.

**How to cite this article:**

Dharmani CKK, Pathak A, *et al.* Prevalence of Traumatic Dental Injuries to Anterior Teeth in 8–12-year-old Schoolchildren of Patiala City, Punjab, India: An Epidemiological Study. Int J Clin Pediatr Dent 2019;12(1):25–29.

## INTRODUCTION

Traumatic dental injuries (TDIs) have become an important public health problem not only because their prevalence is relatively high but also because their treatment has been neglected resulting in a substantial impact on children's oral health-related quality of life. Dental injuries occurring due to sports can be prevented by the use of protective mouth guard or facemask but maximum injuries occur unexpectedly during daily life activities and mostly involve anterior teeth, thus, affecting their function.^1^ Dental trauma is also a source of distress for the parents but they are not aware of the consequences of dental trauma.

A number of risk factors need to be considered when assessing traumatic dental injuries. Incompetent lips and increased incisal overjet are important predisposing factors. Childhood obesity has become a global epidemic. Unhealthy eating habits are one of the causes of obesity in children. Literature shows that the relationship between TDIs and obesity has been investigated in various studies. Petti et al.^2^ and Nicolau et al.^3^ have found that obese children are less active and lethargic and, thus, more prone to dental trauma. But some authors have the opinion that there is no significant association between obesity and TDIs.^4–6^ The association of dental trauma with obesity is not yet clear. Other prevalence studies conducted in Indian children have not investigated the relationship of TDIs with obesity.

The present study was conducted to determine the prevalence of TDIs to anterior teeth in 8–12-year-old schoolchildren of Patiala city in North India and to investigate the relationship of dental trauma with various risk factors.

## MATERIALS AND METHODS

Ethical clearance to carry out the study was obtained from the Ethical Committee of the Institute. The study was a cross-sectional survey carried out on schoolchildren of Patiala city in the state of Punjab in North India. A list of schools was obtained from the District Education Office (DEO) of Patiala. Formal letters were sent to the schools for the examination of schoolchildren explaining the aim and importance of the study and necessary permission was obtained. The principal of the institution was informed about the survey schedule.

The study was carried out on 3,000 schoolchildren aged 8–12 years, equally derived from government and private institutions. A simple random sampling was done. Children of both sexes were examined. Children who had entered 8th or 12th year on their last birthday were included in the study. Children aged 8.0–8.11 years were included in the 8-year age group, 9.0–9.11 years were included in the 9-year age group, 10.0–10.11 years in the 10-year age group, and 11.0–11.11 years in the 11-year age group. A sufficient number of mouth mirrors, probes, tweezers, instrument trays, gloves, masks, and gauze were packed and sterilized for each day of work. Only one examiner investigated all the children to avoid interexaminer variability. The child was seated on a chair in front of the examiner in a manner that ensured proper visualization of the oral cavity in adequate daylight. Name, sex, age, date of birth, and name of school were noted on the proforma.

The body mass index (BMI) for each child was calculated using the formula (BMI = kg/m^2^). Children were weighed (in kilograms) on a weighing machine. The height was measured (in meters) using height charts. BMI for specific age and sex was evaluated on the basis of BMI for age-percentile charts developed by the National Center for Health Statistics (NCHS) in collaboration with the National Center for Chronic Disease Prevention and Health Promotion (CDC).^7^ BMI for age was classified according to the Indian Association of Paediatrics, Ghai OP et al.^8^ Underweight: BMI for age less than 5th percentile. Healthy weight: BMI for age between the 5th percentile and 85th percentile. Overweight: BMI for age between the 85th percentile and 95th percentile. Obese: BMI for age equal to or greater than the 95th percentile.

Clinical examination included the examination of lip competency, incisal overjet, overbite, and molar relationship. Determination of lip competency was done with the child seated on a chair without his/her awareness. Incisal overjet was measured with a ruler by measuring the horizontal distance (in mm) parallel to the occlusal plane between the incisal edge of the most labial maxillary central incisor and the labial surface of mandibular central incisor while in centric occlusion.^9^ Overbite was measured on the mandibular central incisor from the incisal edge to a point on the labial surface perpendicular to the projection, on this surface, of the incisal edge of the maxillary central incisor. It was measured in mm.^10^ The anterioposterior molar relationship was recorded according to Angle's classification.

A detailed history of injury was taken regarding various parameters like time elapsed since injury, place, and cause of injury. Dental injuries were recorded according to the Ellis classification.^11^ Visual inspection and digital examination were done to assess fractured teeth. Discolored teeth and teeth associated with fistulous tract were noted as non-vital. Root fractures were not recorded as radiographs were not taken. Pulp vitality testing was not included in the study. The class 9 fracture was not included as children were 8–12 years old and permanent incisors had erupted.

Statistical analysis was done using Statistical Package for Social Sciences (SPSS) version 16.0. Descriptive analysis was done (frequency distribution and cross-tabulation). The Chi-square test was used to compare qualitative data and determine the statistical significance. The level of significance was set at *p* ≤ 0.05.

## RESULTS

**Fig. 1:**
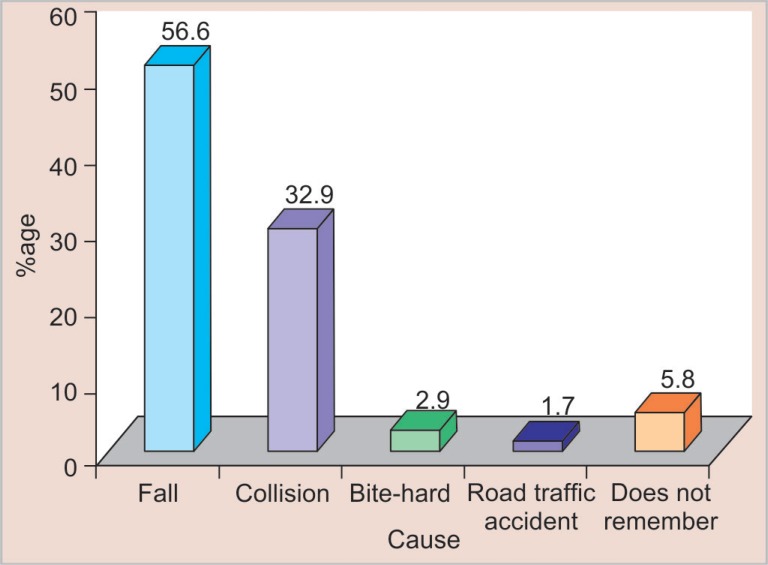
Distribution of traumatic dental injuries according to cause of injury

**Fig. 2:**
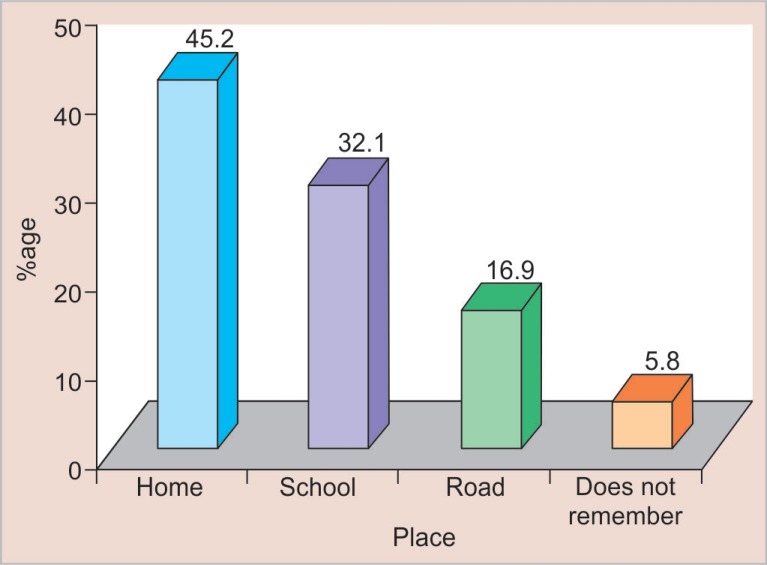
Distribution of traumatic dental injuries according to place of injury

Out of 3,000 schoolchildren examined, 343 (11.4%) children were found to have TDIs to permanent anterior teeth. Table 1 shows the prevalence of traumatic dental injuries according to age, sex, and type of institution. Maximum children with dental trauma belonged to the 11-year age group (19.1%). Male children (14.5%) sustained more injuries as compared to female children (8.4%). The prevalence of dental trauma was more in government institutions (13.3%) as compared to private institutions (9.5%). A maximum number of TDIs occurred due to fall (Fig. 1) with the home being the most common place of injuries (Fig. 2).

**Table 1:** Prevalence of TDIs according to age, sex and type of institution

*Variables*	*No. of children examined*	*No. and % of children with TDIs*	p*
Age			
8 years age group	750	45 (6)	<0.001
9 years age group	750	63 (8.4)	
10 years age group	750	92 (12.3)	
11 years age group	750	143 (19.1)	
Sex			
Male	1,500	217 (14.5)	<0.001
Female	1,500	126 (8.4)	
Type of institution			
Government school	1,500	200 (13.3)	<0.001
Private school	1,500	143 (9.5)	

* Chi square test

Table 2 shows the prevalence of traumatic dental injuries according to the characteristics of obesity, lip competency, incisal overjet, and molar relationship. Among 3,000 schoolchildren examined, 103 children were found to be obese. The prevalence of TDI in children who had BMI for age less than 95th percentile was found to be 11.5% as compared with the prevalence of 10.7% in children who had BMI for age greater than the 95th percentile (i.e., obese). The relationship between obesity and TDIs was found to be statistically insignificant (*p* = 0.807, *p* > 0.05). Children with incompetent lips experienced more dental trauma (37.4%) as compared to children with competent lips (6.7%) (*p* < 0.001). There was also a significant association between dental trauma occurrence and incisal overjet ≥5 mm (*p* < 0.001). Children with Angle's class II div 1 molar relation had maximum TDIs as compared to children with Angle's class I, class II div 2, and class III molar relation (*p* < 0.001).

**Table 2:** Prevalence of TDIs according to characteristics of obesity, lip competency, incisal overjet, and molar relationship

*Variables*	*N*	*No. and % of children with TDIs*	p*
BMI for age of children			
<95th percentile	2,897	332 (11.5)	>0.05
>95th percentile (obese)	103	11 (10.7)	
Lip competency			
Competent lips	2,538	170 (6.7)	<0.001
Incompetent lips	462	173 (37.4)	
Incisal overjet			
≤5 mm	2,636	189 (7.2)	<0.001
≥5 mm	364	154 (42.3)	
Molar relationship			
Class I	2,557	264 (10.3)	<0.001
Class II div 1	264	69 (26.1)	
Class II div 2	142	8 (5.6)	
Class III	37	2 (5.4)	

* Chi square test. *N*: number of children presenting with the variable

**Fig. 3:**
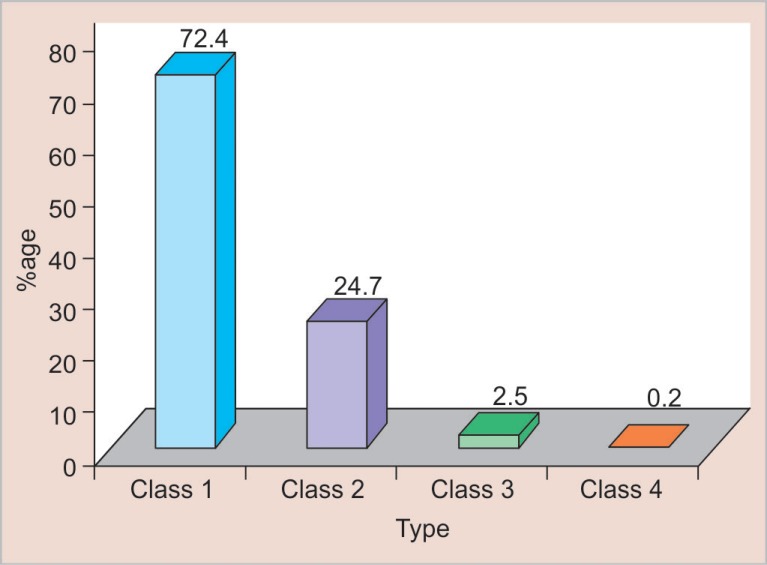
Distribution of traumatic dental injuries according to type of trauma (Ellis classification)

Most of the TDIs involved maxillary teeth (92.4%). Maxillary central incisors were most commonly involved (83.8%) followed by maxillary lateral incisors (8.3%), mandibular central incisors (5.3%), mandibular lateral incisors (2.3%), and maxillary canine (0.2%). Most of the children who experienced dental trauma had only one tooth traumatized (76.1%), two teeth were traumatized in 22.1% children, three teeth in 1.5% children, and four teeth in only 0.3% children. The Ellis class I type of trauma was the most predominant type (Fig. 3).

## DISCUSSION

Dental injuries may have an impact on children's quality of life as a consequence of pain, loss of function, and poor esthetics. Children who are exposed to trauma are not only physically but also psychologically affected. In the present study, the prevalence of TDIs to permanent anterior teeth was found to be 11.4%. This was similar to the prevalence reported by Ingle et al. (11.5%)^12^ and Marcenes et al. (11.7%).^13^ The prevalence was found to be more when compared with David et al. (6.1%),^14^ Tangade (4.41%),^15^ and Gupta et al. (4.15%)^16^ and less when compared with studies done by Ravishankar et al. (15.1%)^17^ and Gupta et al. (13.8%).^18^ Differences in the type of study, classification of dental trauma, age groups, and behavioral differences between different countries may affect the prevalence obtained in various studies.

Maximum children with dental trauma belonged to the 11-year age group (19.1%). The prevalence was found to increase with increasing age which may be attributed to the cumulative effect of sustained injuries over a period of time. This indicates accumulated treatment needs due to lack of dental awareness as seen in various other studies.^1,13,19^ Male children sustained more injuries as compared to female children in the ratio of 1.7:1. Boys are more engaged in leisure activities or sports, take greater risk, are more robust and aggressive in nature, love outdoor activities, and, thus, are more prone to trauma. The prevalence of dental trauma was more in government institutions as compared to private institutions. This has been reported in several other studies.^20,21^ The possible reason might be that children from government institutions usually belong to less educated and poor families and they are left unattended by their parents. A number of children per family are also more with consequent less attention being paid to each child.

Fall (56.6%) was the most common cause of TDIs followed by collision (32.9%). About 5.8% children did not remember the cause of injury especially when the traumatic injury involved only enamel. The most common place where dental injuries occurred was home (45.2%) followed by the school (32.1%) as reported in previous studies.^17,22^ Children spend most of their time at home followed by school where they are engaged in various activities like bicycle riding, fighting with siblings, etc. Games during lunch breaks in school can cause trauma due to fall, fighting among friends. It is important that preventive education should be given to parents, children, and school teachers. Physical sports activity at school should be supervised by the sports teachers all the time and appropriate preventive measures should be made compulsory, i.e., helmet, protective mouth guard, and face mask.

Childhood obesity is evolving as a major nutritional problem in developing countries due to various nutritional, socioeconomic, and behavioral transitions. Children nowadays eat more junk, unhealthy food with less, or no physical activity contributing to the rapidly rising prevalence of obesity. Childhood obesity should be prevented since it is a risk factor for many diseases. In studies conducted by Petti et al.,^2^ Nicolau et al.,^3^ and Soriano et al.,^21^ it was reported that obesity significantly increased the risk of TDIs. According to these authors, obese children present less agility, are less active, lethargic, and clumsy and, thus, are more prone to trauma. In India, the relationship between obesity and dental trauma has not been investigated. This study included obesity as one of the factors affecting trauma and the association between obesity and dental trauma was found to be statistically insignificant. This is similar to studies done by Tapias et al.^6^ and Soriano et al.^5^ More studies are required to be done to determine the association between obesity and dental trauma.

Children with incompetent lips experienced more dental trauma (37.4%) as compared to children with competent lips (6.7%). There was also a significant association between dental trauma occurrence and incisal overjet ≥5 mm. With the increase in overjet maxillary incisors become more protrusive making them more prone to dental trauma. Also, the reduction of cushioning effect in the presence of incompetent lips predisposes the teeth to trauma. Children with Angle's class II div 1 molar relation had maximum traumatic dental injuries as compared to children with Angle's class I, class II div 2, and class III molar relation. This could be due to increased overjet because of protruded maxillary incisors and incompetent lips. Trauma may be prevented if orthodontic treatment is initiated at an early stage.

Most of the TDIs involved maxillary teeth (92.4%). This is similar to earlier findings.^17^ Maxillary teeth are generally more proclined and tend to be the first to receive a direct blow resulting in trauma. Also, the upper jaw is fixed to the skull which makes it rigid. The lower jaw tends to reduce the impact forces directed on the lower anterior teeth by movement. Most common teeth to get traumatized were maxillary central incisors followed by maxillary lateral incisors which corroborates the findings of other studies.^12^ Maxillary central incisors hold a prominent and vulnerable position in the arch and are frequently protruded which make them more prone to trauma. Maxillary left central incisors were more commonly involved as compared to maxillary right central incisors.

Most of the children had only one tooth traumatized (76.1%). When one tooth is traumatized, the majority of the force of impact is dispersed by the fractured tooth and no more teeth are traumatized. Ellis class I type of trauma was the most predominant type. This was in accordance with other studies.^17^ Enamel is the outermost hard covering of the coronal part of the tooth which gives protection to underlying tissues. So it is frequently fractured while facing direct trauma. Also, children with fracture involving enamel only generally do not have any complaint and sometimes they are not aware of their traumatized teeth. For this reason, the percentage of fracture involving only enamel might be higher in this study.

## CONCLUSION

- The prevalence of TDIs in the present study was found to be 11.4%. Maximum injuries occurred in the 11-year age group (19.1%) and were more in boys (14.5%) than girls (8.4%). Children of government schools sustained more dental injuries (13.3%).
- Fall was the most common cause (56.6%) and the home was the most commonplace (45.2%) of dental trauma in children.
- The present study has shown no significant relationship of TDIs with obesity but significant association was seen between dental trauma and incompetent lips, incisal overjet ≥5 mm and Angle's class II div I molar relation.
- Most common teeth to get traumatized were maxillary central incisors (83.8%). 76.1% of children had only one tooth traumatized. The most predominant type of trauma was the Ellis class I (72.4%).

## CLINICAL SIGNIFICANCE

Although the results of this study have shown that there is no significant relationship of TDIs with obesity in 8–12-year-old schoolchildren of Patiala city. The prevalence of childhood obesity is increasing and there is a need for more studies to be conducted to investigate childhood obesity as a possible risk factor for dental trauma. The study has also revealed that the majority of the TDIs remain untreated which indicates negligence and low priority given to dental health. There is a need for greater awareness among children and parents regarding dental trauma.
